# Effects of Residence Time, Auto-Fertility and Pollinator Dependence on Reproductive Output and Spread of Alien and Native Asteraceae

**DOI:** 10.3390/plants8040108

**Published:** 2019-04-23

**Authors:** Anna Corli, Christine S. Sheppard

**Affiliations:** 1 Institute of Landscape and Plant Ecology, University of Hohenheim, August-von-Hartmann Str. 3, 70599 Stuttgart, Germany; anna.corli01@universitadipavia.it; 2 Department of Earth and Environmental Sciences, University of Pavia, Pavia, Via S. Epifanio 14, 27100 Pavia, Italy

**Keywords:** alien–native species continuum, Asteraceae, auto-fertility, multi-species experiment, plant invasion, pollinator dependence, residence time

## Abstract

Alien plants benefit from auto-fertility to spread over areas where the lack of co-evolved mutualists would otherwise limit invasion success. However, the widespread generalists among mutualists and their large geographical ranges allow alien plants to be integrated into networks. The role of residence time also has to be accounted for, as it takes time for a species to spread and adapt to a new area. We investigated how residence time, auto-fertility and pollinator dependence affect reproductive output and invasion success of Asteraceae in Germany. We conducted a multi-species common-garden experiment along an alien–native continuum including 42 species of natives, archaeophytes and neophytes (casual and established), subjecting plant individuals either to free access or exclusion of pollinators. Pollinator dependence does not play a crucial role in invasion success, with most Asteraceae being able to self-fertilize. Surprisingly, both established neophytes and natives showed higher abilities to self-fertilize, while archaeophytes and casual neophytes were more attractive to pollinators. In contrast to casual neophytes, the established neophytes’ strategy was associated with a large reproductive output. Yet, auto-fertility was not associated with range size, since archaeophytes reached the largest range sizes. Elucidating how breeding systems affect invasion success is crucial for predicting and managing invasions.

## 1. Introduction

Across the globe, thousands of plant species, called “aliens”, have deliberately or accidentally been introduced to biogeographic regions where they are non-native. In fact, at least 3.9% of species in the global flora, that is more than 13,000 species, have now established self-sustaining populations in regions where they did not occur naturally [1]; some of them have also become “invasive” through the expansion of their range in new regions [2], threatening native communities and ecosystems.

Successful plant invasions depend on the interactions between alien species and the native recipient community [1]. In this context, several hypotheses have been proposed in invasion biology on how alien species can successfully establish and spread in new habitats despite their lack of local adaptations. For instance, the enemy-release hypothesis posits that alien species colonize new areas because they escape their natural enemies: On introduction to a new region they experience a decrease in regulation by herbivores and other enemies, which can lead to a rapid increase in abundance and range size [3]. On the other hand, when moved to a new environment alien plants also leave behind their mutualists, such as their common pollinators; changes in mutualist species composition may limit or even prevent invader success if the pool of mutualists available to an alien plant does not include species on which it has evolved to depend [4]. Thus, for the establishment and the success in non-native regions, alien plants may experience different breeding system adaptations compared with natives, such as auto-fertility [2]. 

In the context of invasion biology, plants that can self-fertilize and are self-compatible should be better colonists than those that cannot, as stated by one of the major botanists of the last century, Herbert G. Baker. This is because auto-fertility would allow single individuals, isolated from suitable mates and pollinators by long distance dispersal, to found new populations [5]. As a trait of invasiveness in alien flora, auto-fertility can increase plant performance: Autogamous plants experience higher capacity for autonomous fruit and seed production than congeneric species that have not become naturalized [6,7,8] and higher fecundity than natives [9]. However, there could also be negative effects post-reproduction, resulting in lower seed germination success, inbreeding depression and lower seed quality: Although auto-fertility allows plants to transmit two copies of their genome to their seed progeny whereas outcrossed plant contribute only one, with a substantial potential fitness advantage [10], auto-fertility is also expected to decrease heterozygosity, resulting in the expression of deleterious recessive alleles and an increase in inbreeding depression [11]. Nevertheless, auto-fertility has been demonstrated to have a positive correlation with the size of the invaded range, with larger positive effects on range size for abiotically pollinated species than biotically pollinated ones [7,8,12]. 

However, auto-fertility may not be the only successful breeding system for alien plants. The widespread host generalism among plant mutualisms and the large geographical ranges of many mutualists [2] suggest that there might be insufficient variation in mutualist species composition among comparable plant species for mutualist composition to influence invader success [13]. Therefore, as already demonstrated by several studies, alien plants could also be visited in the novel range by generalist pollinators, where they seem also well integrated into the plant–pollinator networks [2,12]. Although few studies have addressed the potential impacts of generalist pollinators on invasions, McIver et al. [14] demonstrated that *Apis mellifera* (honeybee) are able to facilitate the invasion by aliens, specifically *Centaurea solstitialis*, by increasing seed set compared to that achieved by native pollinators alone. Moreover, even if alien plants appear poorly integrated into plant–pollinator networks, they may still achieve high pollination service and high reproductive success [15], with only a small proportion known to be prevented from spreading because of the absence of pollinators [12]. Therefore, the success and the establishment in a new environment for alien plants could be guaranteed either due to their capacity to attract pollinators or due to auto-fertility or even both. Overall, there are conflicting results on whether there are differences in the frequency of pollination modes between native and alien species. A study in Great Britain found that aliens were more likely insect-pollinated than native plant species [16], whereas a study on North American flora concluded that they were not [17], while a study in Germany showed that 62% of natives and only 51% of alien were insect-pollinated [18].

Functional traits and interactions between invader and native residents are not the only causal factors that explain invasion dynamics, but also stochastic effects, residence time or the number of introduction events (propagule pressure) [19]. Indeed, one of the most supported generalizations in invasion biology attests that the probability of establishment increases with time since the introduction of a species to a new area, that is its residence time [20]. Native plants and archaeophytes, that is non-native species which were introduced outside their original range before Columbus’ discovery of America (1492), are more common and have larger range sizes than neophytes, which arrived later [20]. Residence time has been shown to also be more crucial than selected species traits in explaining alien species distributions [21,22,23]; a longer residence time provides species with more time to accumulate and release propagules, and to disperse in a region and thus may increase a species’ chance to establish self-sustaining populations in the wild [19]. Furthermore, with longer residence times an alien plant may adapt to its new environment. 

To shed light on the roles of auto-fertility, pollinator dependence and residence time on invasion success, this paper reports an experimental study on the effects of flower visitors (potential pollinator and other insects) exclusion on reproductive output of Asteraceae plants characterized by varying residence times, using an alien–native species continuum (including casual and established neophytes, archaeophytes, natives, hereafter referred to as invasion status groups). Casual neophytes thereby refer to alien plants that occasionally occur in the wild but have not established self-sustaining populations [20]. As many eco-evolutionary processes relevant to invasions are expected to change gradually over time, the status of an immigrant species may be better described by continuous residence time [23]. Yet, differences among invasion status groups may be relevant due to an introduction bias leading to differences in species characteristics [23]. To elucidate such changes over time and differences between invasion status groups, a comparison of multiple species growing at the same location is required [1,24], although multi-species experiments are still rarely used in ecology despite their usefulness in searching for general patterns and mechanisms [25].

Spanning a wide range of minimum residence times (MRT) and covering all invasion status groups, plant individuals of each of 42 annual Asteraceae species (Supplementary Materials Table S1) were either isolated from or had free access to flower visitors aiming to evaluate, how reproductive output might be influenced by invasion status, residence time of the species and the presence/exclusion of flower visitors, and whether this has an influence on species’ range sizes. In particular, these outstanding questions were addressed: (i) Does the number of flower visitors depend on residence time or invasion status of species? (ii) How does invasion status of the species influence the reproductive output of Asteraceae species in presence/exclusion of flower visitors? (iii) To what extent, if at all, is seed germination influenced by the presence/exclusion of flower visitors? (iv) What is the effect of residence time and auto-fertility on range size? We hypothesize that the number of flower visitors increase with residence time and hence length of co-evolutionary history, thus natives are more frequented by flower visitors than aliens. Hence, neophytes have a lower pollinator dependence (higher auto-fertility respectively). We expect seed germination success to be lower in absence of potential pollinators, and finally that longer residence time and higher auto-fertility increase range sizes.

## 2. Results

### 2.1. Effects of Residence Time and Invasion Status on Flower Visitors

**Figure 1:**
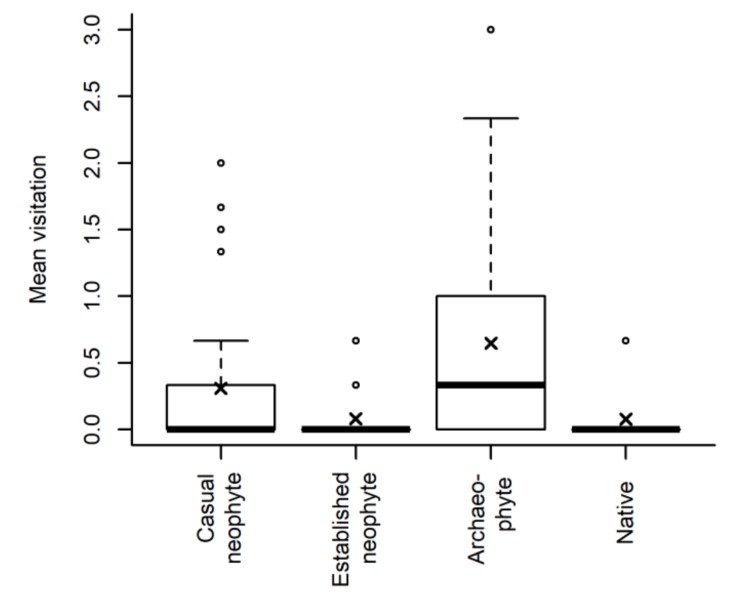
Number of flower visitors (mean of three days of observation) depending on invasion status (for 31 species and 100 individuals that had flowers open at the time of flower visitor observation). The boxplots show the median per group (solid line), crosses the mean, the boxes represent the 25th and 75th quantile, whiskers the normal data range and circles the outliers.

Nine species never flowered during the experiment (*Centaurea diffusa*, *Erigeron annuus*, *Erigeron canadensis*, *Erigeron sumatrensis*, *Iva xanthiifolia*, *Lactuca serriola*, *Lactuca virosa*, *Lapsana communis*, *Rudbeckia hirta*) while two species (*Senecio vulgaris* and *Sonchus asper*) had already finished their flowering time before flower visitors were observed. For the remaining 31 species, we found no effect of MRT on flower visitors (likelihood ratio test, χ^2^ (1 df) = 0.22, *p* = 0.637). However, there is a significant effect of status (likelihood ratio test, χ^2^ (3 df) = 10.67, *p* = 0.014) with archaeophytes receiving the most flower visitors (Figure 1). Of note, flower visitors (potential pollinators) that visited plants were mainly honeybees (*Apis* spp.), bumblebees (*Bombus* spp.) and flies (Diptera); whereby archaeophytes received the most fly visits and individuals of this status group were also on average more often visited by bees (Supplementary Materials Table S2).

### 2.2. Effects of Pollinator Exclusion and Invasion Status on Reproductive Output 

Coincidentally there were significant differences in plant height at the beginning of the experiment, with the “without pollinators” treatment (mean 3.9 cm) having slightly smaller initial heights compared to the “with pollinators” treatment (mean 4.3 cm, likelihood ratio test χ^2^ (1 df) = 11.45, *p* < 0.001, Supplementary Materials Figure S1). Nevertheless, plants in the “without pollinator” treatment reached higher final height (Table 1, Figure S1). 

**Figure 2:**
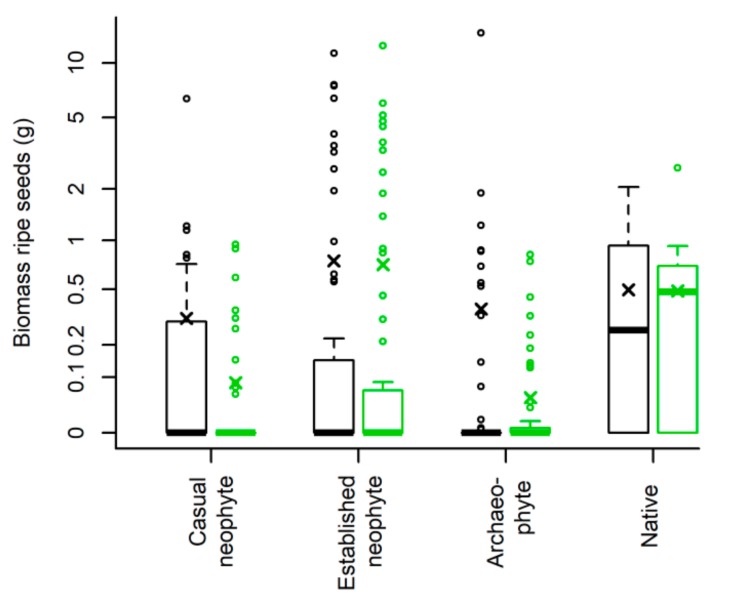
Ripe seeds biomass depending on invasion status for the 42 Asteraceae species (397 individuals). Treatment “with pollinators” in black, treatment “without pollinators” in green. The boxplots show the median per group (solid line), crosses the mean, the boxes represent the 25th and 75th quantile, whiskers the normal data range and circles the outliers. Axis is shown on a log-scale.

Depending on invasion status, biomass of ripe seeds differed between treatments (marginally significant interaction, Figure 2, Table 1): While there were no differences in seed biomass between treatments for natives and established neophytes (both mean native “with pollinators” and “without pollinators”: 0.49 g; mean established neophytes “with pollinators”: 0.75 g; mean established neophytes “without pollinators”: 0.71 g), for archaeophytes and casual neophytes the treatment “with pollinators” (mean archaeophytes: 0.37 g; mean casual neophytes: 0.32 g) reported a higher seed biomass than the treatment “without pollinators” (mean archaeophytes: 0.05 g; mean casual neophytes: 0.09 g).

Similarly, there was a marginally significant interaction between invasion status and treatment for the number of ripe seeds (Table 1, Figure S2): Established neophytes, which overall had the highest number of ripe seeds (with a mean of 3239) and natives reported a higher number of ripe seeds in the treatment “without pollinators”, whereas archaeophytes and casual neophytes showed the opposite trend, with a higher number of ripe seeds in the treatment “with pollinators”. Overall, 21 of 42 species produced ripe seeds in the treatment “with pollinators” compared to 23 in the treatment “without pollinators” (Supplementary Materials Table S1), of which three species only produced seeds in the treatment “without”. 

However, there were no differences among treatments, invasion status or their interaction in total reproductive biomass and number of capitula (Table 1). Furthermore, no differences were seen among treatments or invasion status in the day of first flowering, yet there were differences among status in the first day of seed ripening (Supplementary Materials Figure S3), where natives produced seeds the earliest (mean 59 days), followed by archaeophytes (mean 66 days), established neophytes (mean 69 days) and casual neophytes (109 days), which produced seeds only towards the end of the experiment.

### 2.3. Effects of Pollinator Exclusion on Seed Germination

**Figure 3:**
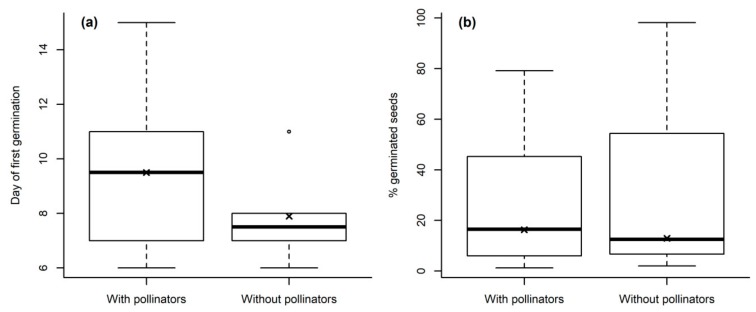
(**a**) Day of first germination; and (**b**) percentage of germinated seeds for seeds collected from the two treatments (for ten species that germinated in both treatments only). The boxplots show the median per group (solid line), crosses the mean, the boxes represent the 25th and 75th quantile, whiskers the normal data range and circles the outliers.

Surprisingly, seeds from the treatment “without pollinators” germinated marginally significantly earlier than plants where flower visitors had free access (likelihood ratio test, χ^2^ (1 df) = 3.53, *p* = 0.060, Figure 3a). However, there were no differences in the percentage of germinated seeds between treatments (likelihood ratio test, χ^2^ (1 df) = 1.60, *p* = 0.207) (Figure 3b). 

Plants with the highest germination rates were *Bidens pilosa*, *Carthamus lanatus* and *Carthamus tinctorius* that reported respectively 61.1%, 64.4% and 72.9% of germinated seeds (Supplementary Materials Table S3). Note that only ten species that produced ripe seeds in both treatments were included in this analysis, half of which were casual neophytes (five species).

### 2.4. Effects of Residence Time and Pollinator Dependence on Range Size

**Figure 4:**
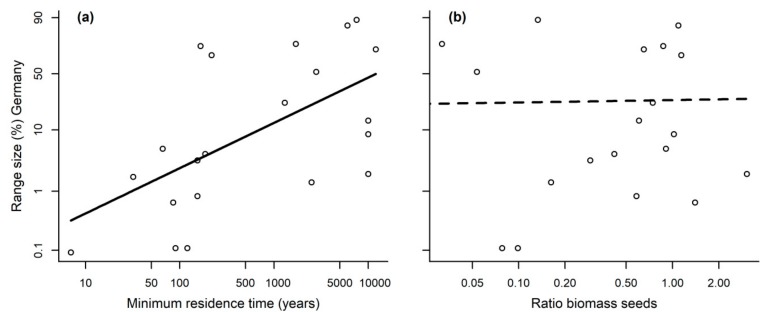
(**a**) Range size depending on minimum residence time; and (**b**) pollinator dependence (i.e., measured as mean biomass seeds without pollinators/mean biomass seeds with pollinators) for the 21 Asteraceae species for which the ratio could be calculated (i.e., biomass seeds with pollinators not zero). All axes are shown on a log-scale. Significant effects (with the other covariate fixed at its mean) shown with a solid line, non-significant effects with a dashed line.

MRT had a significant positive effect on range size in Germany (F (1,18 df) = 9.875, *p* = 0.006, Figure 4a). Yet, pollinator dependence (measured as the ratio in total seed biomass without pollinators versus with) had no significant effect on range size in Germany (F (1,18 df) = 0.012, *p* = 0.915, Figure 4b). 

A significant effect of invasion status on range size in Germany (F (3,38 df) = 10.401, *p* < 0.001) revealed that archaeophytes have the largest and casual neophytes the smallest range sizes (Supplementary Materials Figure S4).

## 3. Discussion

We conducted a multi-species common-garden experiment along an alien–native species continuum, testing the roles of residence time, pollinator dependence and auto-fertility on reproductive output and invasion success (range size) of Asteraceae plant species. Breeding systems play a crucial role in the establishment in the new environment. In a global change scenario, comparisons between native and alien species with varying residence time (reflecting varying lengths of local adaptation and co-existence with native communities) are important to understand mechanisms driving invasion success [23], while “invasion status” may provide information about differences in species characteristics due to an introduction bias. We here show that auto-fertility with no apparent reduction in seed quality is associated with high reproductive output of established neophytes and natives. Nevertheless, for plants to be successful at the large scale, auto-fertility is not necessarily important, with archaeophytes—which were most frequently visited by insects (and thus probably pollinators)—reaching the largest range sizes.

### 3.1. Effects of Residence Time and Invasion Status on Flower Visitors

Generally, the number of insects visiting the flowers was low: The most visited plant, the archaeophyte *Anthemis arvenisis*, reported an average of three visits among five replicates. Moreover, some plants did not even produce flowers and others mainly flowered at the end of the field season. Due to this delayed flowering, which may have resulted from transplanting shock, flower visitor observations were only assessed at the end of September and October, and the five-minute intervals may have been too short. In addition, the location of the common garden adjacent to arable land, ruderal vegetation and intensively used grasslands may also explain the low number of visitors.

Nevertheless, for the flowering species that we could observe, residence time did not affect the number of flower visitors while invasion status did. This suggests that rather than specialist visitors gradually adapting to new immigrant species, a priori differences among invasion status groups caused by an introduction bias may be more relevant. Archaeophytes and casual neophytes received the most insect visits, with archaeophytes having the highest number. These groups include most species that produce flowers with larger diameters. Our results support the assumption that alien species are pollinated in their new ranges by generalist insects [26]; generalized pollination system increases thus plants’ likelihood to attract generalist pollinators and to readily establish new mutualistic interactions [27]. Conversely, established neophytes and especially natives did not attract as many flower visitors as archaeophytes and casual neophytes did, an outcome in line also with the results on the treatment and status effect on seed biomass and seed number reported below.

There is a disproportionally high representation of pollination by insects in early stages of invasion, followed by shifts progressively to auto-fertility as species become naturalized or invasive, as already noted by Pyšek et al. [12]. This scenario is the same as was noted for the Czech flora, where alien species introduced to central Europe contained a higher proportion of insect-pollinated species than did the central European native flora [12]. Archaeophytes and casual neophytes seemed thus well-integrated into plant–pollinator networks where flower visitors have visited them as already noted by other studies on alien plants [2,12].

If results for established neophytes were expected according to the theory of invasiveness [5], the results for natives were more surprising. This could be due to the fact that natives tended to have fewer open flowers during the time of our visitor observations (although flowering time did not differ among invasion status groups), which may have affected the results. Nevertheless, assuming that natives were less visited because less reliant on pollination mutualisms, studies demonstrated that more than half of outcrossing species permit self-pollination to some degree [28].

### 3.2. Effects of Pollinator Exclusion and Invasion Status on Reproductive Output

Our experiment showed that many natives, archaeophytes, casual neophytes and established neophytes were auto-fertile to some degree. Indeed, exclusion of flower visitors did not significantly affect many of our reproductive performance measures, suggesting that pollinator dependence may not play a crucial role in the spread of alien plants. This result was already noted [29] and could partially be explained as plants used in our experiment were annuals. Indeed, auto-fertility is thought to be more common in annual compared to perennial plants because annuals have a lower genetic load and suffer less from inbreeding depression [30]; moreover, it provides the demographic benefit of reproductive assurance during an annual plant’s only opportunity for reproduction [31]. In addition, as already demonstrated for other flowering plants, plant species in central Europe reported a low degree of competition for pollinators compared to other parts of the globe, as a consequence of pollen limitation [32], in line with the less diverse temperate floras compared to more diverse tropical floras [33]. In particular, plants evolve traits that reduce reliance on pollinators like auto-fertility and even shifts in flowering time [32]. Therefore, the observed auto-fertility and the low levels of pollinator dependence might not be specific to our species necessarily, typical for a central European semi-natural landscape, but might be common in other parts of central Europe as well, as already noted by Razanajatovo and van Kleunen [29]. 

Our results attested that plants’ number of capitula, reproductive biomass as well as day of flowering were not different indiscriminately by the presence or the absence of flower visitors; however, difference between treatments were seen in plants with regard to the day of producing seeds as well as the number and biomass of ripe seeds, which represent the closest fitness proxy for annual plants. Natives produced seeds the earliest, followed by archaeophytes and established neophytes. Notably, there are only few studies that tested the role of seed phenology on invasion biology, while most of them focus on flower phenology and its duration [26]. However, our outcome is in line with a previous study by Lediuk et al. [34], where it demonstrated a delay in producing seeds for alien species, with natives being faster. The experienced gap between natives and aliens in seed phenology is explained as an adaptation of the latter species: When flowering or, in our case, seed phenology of the species does not overlap, the lack of resource competition may enhance seed dispersal by mutualists of alien species, thus favoring their spread [2,35,36]. In this way, delaying seed production, alien plants could potentially be faced with an empty niche and thus have the possibility to create new adaptation to new mutualisms. Nevertheless, the very late seed ripening for casual neophytes may be an important factor preventing their invasion success. 

Established neophytes and natives had low reliance on resident flower visitors, thus were more likely to self-fertilize. Indeed, no differences were seen between treatments in biomass of ripe seeds, where plants reported roughly the same amount; notably, established neophytes had the highest ripe seed biomass and, particularly when flower visitors were excluded, the number of ripe seeds was higher, with the highest value for established neophytes. As already demonstrated in several studies, alien plants experience a higher investment in reproduction [37], with a trait of invasiveness associated to smaller and lighter seeds [38], often combined with higher seed production [29]. Several recent studies found evidence that the capability for auto-fertility benefits the establishment, persistence and spread of the species [6,7,8,12] especially when introduced into a new habitat, because it provides reproductive assurance to individuals that have not integrated themselves into resident plant–pollinator networks [2] or in an environment in which suitable mates and pollinators are scarce [5,39,40]. Nevertheless, even if several studies demonstrated the opposite, with alien species being more auto-fertile than natives [8], Burns et al. [41] noted the capability for native species to be prone to self-fertilization, thus supporting our results.

On the contrary, archaeophytes and casual neophytes depend more on flower visitors: Their seed biomass and, similarly, their number of ripe seeds were much higher in the treatment where flower visitors had free access. Thus, archaeophytes differed from neophytes and natives in the frequency of pollination modes used by the species favoring pollination, as already noted [7,8,15,17]. Moreover, the low seed mass of archaeophytes when pollinators were excluded could also suggest that some of the harvested cypselae could have been empty or had aborted seeds, strengthening the link with pollinator-dependence.

### 3.3. Effects of Pollinator Exclusion on Seed Germination

Seed germination is one of the most important stages in the life cycle of plants together with flowering and pollination, fruiting and seed dispersal [42] and it plays an important role in biological invasions [43]; it is considered one of the important phenological stages that are influenced by environmental factors, with abundance and timing determining plant establishment and recruitment [44]. Our results reported that seed germination depended on treatments: Surprisingly, seeds produced by plants when flower visitors were excluded germinated earlier, although the percentage of germinated seeds in the end did not differ. Notably, it has been demonstrated that early germination increases plant fitness [45] through space pre-emption and immediate access to limited resources and it is strongly related with the establishment of plants, enhancing opportunities for invasion success [46].

### 3.4. Effects of Residence Time and Pollinator Dependence on Range Size

Residence time is an important determinant of the geographical range sizes of alien plant species: As also demonstrated in our study, the longer a species has been present in a new area, the greater is its probability to be widespread [20,47,48,49,50,51]. Notably, even if it has been demonstrated that the range size of alien plants is on average smaller than native range size, because most aliens are likely to be still increasing their range, with neophytes at their early stages of invasion not having had enough time to reach their potential spread [21,52], our study highlighted a higher range size for archaeophytes compared to the other range sizes (which was also the case for a larger species set of Asteraceae in Germany) [23]. A comparative study conducted by Williamson and other researchers [53] demonstrated that archaeophytes, many of which have been in western and central Europe for thousands of years, are in equilibrium with their environment and they even have larger range sizes, mainly because of their habitat requirements. Indeed, they are primarily arable weeds especially of warmer soils and loess soils [22].

Nevertheless, pollinator dependence did not show any effect on range size. It has been demonstrated that factors explaining alien species invasion differ in several stages, that is during “transport”, “colonization”, “establishment” and “spread” [54]. In a study on factors that affect alien species success during these stages of invasion [55], it has been shown that traits such as auto-fertility and fast growth, which are typical of the colonization stage, help plants to better colonize a new environment. Conversely, mutualisms with pollinators and seed dispersal agents necessary to ensure the establishment of plants [2], appear in the establishment stage, with seed dispersal agents and thus the capacity to reach larger range sizes more important during spread. It is thus possible that the ability to self-fertilize may play a role for different stages of invasion success, although it is not important for our measure of large-scale success. 

### 3.5. Study Limitations

Overall, we did not find a high degree of pollinator dependence in our study, however there are some limitations to our findings that need to be addressed. Firstly, we only investigated pollinator dependence in one local environment. The relatively low number of flower visitors we observed could thereby be related to the environment: A recent study in Switzerland demonstrated that the frequency of flower pollination and visitation to native and alien plants were lower in garden environments than in natural environments [56]. We cannot be sure whether pollen limitation was an issue, as we did not consider an additional supplemental outcross treatment [31]. As we did not control for the phylogenetic correlation structure, differences among invasion status could be influenced by certain lineages, corresponding to a specific invasion status, being more self-fertile or having more attractive flowers. However, the four invasion status groups were not clustered in the phylogenetic tree (constructed using Daphne [57], Supplementary Materials Figure S5). Furthermore, some species may never have encountered the local pollinator communities if they do not occur in the study region. Ten of our species (of which six are casual neophytes) are not reported to occur in the near vicinity of the common garden, while another four species (two casual neophytes) occur only ca. 5–20 km away (Supplementary Materials Table S1).

There may have been some unwanted side-effects of using organza socks: For one, plant individuals in the “with pollinators” treatment may also have suffered from herbivory. Indeed, some flowers were eaten in four individuals of the “with pollinator” treatment (two *Galinsoga parviflora*, one *Galinsoga quadriradiata* and one *Glebionis coronaria*), with further individuals suffering from leaf herbivory. Organza socks may also have had some effects on microclimatic conditions, as individuals in the “without pollinators” treatment reached larger final heights and achieved earlier seed germination. Nevertheless, such an effect would not be biased with regards to invasion status or residence time. Additionally, the organza sock may have aided pollination within the plant individual (which could explain the surprising results on seed germination, where however we also note that in this part of the study, we could only consider ten species). Lastly, in the “with pollinator” treatment it is possible that some seeds were lost even with the regular checking and collections. 

## 4. Materials and Methods 

### 4.1. Study System

We focused on Asteraceae plants occurring in Germany as study species. The Asteraceae family is the largest family of flowering plants in the world, characterized by approximately 1620 genera and more than 23,600 species [58]. Except for Antarctica, the Asteraceae family shows a cosmopolitan distribution where these species colonized open arid and semi-arid regions of subtropical and lower- to middle-temperate latitudes, but also mesic montane and oceanic environments [59]. Notably, besides the natives, there are numerous archaeophytic and neophytic Asteraceae species found in Germany. 

Floral and fruit characters are the characteristic traits of this family: The inflorescence is named head or capitulum and consists of a few or large number of sessile flowers composed in a disk-like flower-head, that can be actinomorphic or zygomorphic, hermaphrodite or unisexual; the floral formula is K0,C(5),A(5),G2 and the ovary inferior. Seeds are non-endospermic and are dispersed intact with the cypsela, the fruiting body, mainly through anemochory for hairy pappus seeds or epizoochory, where dispersal units have hooks. Pollination is usually mediated by insects, but anemophily is also present [58].

The evolution of this family started in southern South America, and the early radiation and first migrations occurred to southern Africa and later to Europe, Asia and Australasia, with the pappus allowing seeds to drift on the winds and gain a wider distribution [59]. Species were typically characterized by bearing attractive inflorescences and may have promoted the radiation of insect pollinators that heavily rely on this family to feed and reproduce [60]. In this study, we focused on 42 annual Asteraceae species with different invasion status and varying minimum residence time in Germany, hereafter MRT. Specifically, we used five natives, 13 archaeophytes and 24 neophytes, of which 14 are established and ten casual neophytes, with plants’ MRT ranging from 7 to 12,000 years. Information on invasion status was obtained from the floristic database of the Bundesamt für Naturschutz BfN (www.floraweb.de) and the German Flora Rothmaler [61]. Since only few annual native Asteraceae occur in Germany, it was not possible to divide the number of species among groups more equally. Since the precise time when taxa have colonized a new environment is mostly unknown, Rejmánek [47] proposed the term minimum residence time as an estimator of the introduction date, based on the first record of the species which is determined through analysis of herbarium and collection data [21]. Information on MRT of Asteraceae has been extracted from scientific reports, herbaria records, archaeobotanical findings, standard floras, and online databases e.g., BiolFlor ([62], see [23]). Information on the focal species’ annual life cycle was also determined from BiolFlor [62], whereby four out of the 42 species were indicated to be facultative annuals which can also be biennial (*Centaurea solstitialis*, *Erigeron annuus*, *Lactuca virosa* and *Rudbeckia hirta*). We also determined the focal plants’ range size in Germany as an indicator of large-scale success. Occurrence data was obtained from FlorKart, BfN and NetPhyD Netzwerk Phytodiversität Deutschlands e.V. (www.deutschlandflora.de), documenting species’ occurrence per grid cell of 10 × 6 arc minutes (ca. 11 km × 11 km) divided into four quadrants. We then calculated range size in Germany as the proportion of occupied cells.

### 4.2. Experimental Design

A pot experiment was set up in an experimental garden (48°42′N, 9°11′E, 400 m a.s.l.) at Hohenheim University in Stuttgart, Germany. The climate was oceanic, subtype subcontinental, mild, with no dry season and warm summers; mean annual temperature is reported at 8.5° C and total annual precipitation was attested at 685 mm. While a common garden experiment has the limitation of representing only one local pollinator community, field tests would not be feasible while including so many focal species.

To study the effects of pollinator exclusion and invasion status on reproductive output, the common garden experiment was conducted from July to October 2016: 42 Asteraceae species were used, with five replicates for each of two treatments aiming to assess pollinator dependence (“with pollinators”, representing full access of the natural pollinator community and other insects, and “without pollinators”, completely excluding flower visitors, but also herbivores), giving a total number of 420 pots. Pots of a volume of 2 L (12 × 12 × 20 cm) were set up on wooden planks, where ten labelled pots were randomly arranged in each row, at 20 cm distance from each other. A drip water irrigation system was installed where one needle, placed in each pot, supplied five times per day a total amount of 0.42 L water per day, from 11 am to 6 pm. Within the first two weeks, dead individuals were replaced.

Seeds of the 42 species, obtained both from wild populations in Baden-Württemberg and German Botanical Gardens (Berlin, Berlin-Dahlem, Bonn, Dresden and Hohenheim, Supplementary Materials Table S1), were germinated in seed trays in spring 2016. Thereafter on July 8th plants were transplanted individually into the 2 L pots filled with field soil. For the treatments where flower visitors were excluded, plants were enclosed in an organza sock (50% polyethylene, 50% nylon) before flowering (i.e., a sock around the whole plant, allowing pollen transfer across flowers within the plant individual, but excluding all insects: Pollinators and others). A few seedlings were lost over time due to browsing by foxes and crows, especially those enclosed in organza socks. All remaining plants were harvested either at the end of their life cycle (from October 17th onwards) or at least 15 weeks after the start of the experiment.

To study the effects of pollinator exclusion on seed germination, a greenhouse experiment was set up in November testing the germination success of seeds from the treatment with versus without pollinators. Ideally, 20 ripe seeds of each treatment (fewer if not available) collected from the common garden experiment were sown in ten seed trays (12 × 20 rows) filled with topsoil, where each row was used for seeds from a single individual. We did not cold-stratify the seeds, as we learnt from a prior experiment with the same species that all species are principally able to germinate without such a treatment (Brendel et al., unpublished). We used 162 ripe seeds from 26 different species that successfully reproduced. Every two days, for 28 days, plants were manually watered, and the germination of seeds was monitored.

### 4.3. Data Collection

In the common garden experiment set up to study the effects of pollinator exclusion and invasion status on reproductive output, plant height was measured both at the beginning as well as at harvest of the experiment. Flowering starting date and seed ripening date were checked 3× per week. For plants where flower visitors had free access (“with pollinators” treatment), we manually collected ripe seeds throughout the course of the experiment (checking 3× per week); conversely, for plants enclosed in the organza socks (“without pollinators” treatment), seeds were trapped inside and collected directly at harvest. Plants were harvested by cutting off stems at the soil level; for each individual, final height was measured and total number of capitula was counted and separated from the plant body. Plant material was dried at 70 °C for 72 h; vegetative biomass (stems and leaves), reproductive biomass (capitula and not ripe seeds) and ripe seeds were weighed with a high-precision balance. Total reproductive biomass was obtained by adding ripe seed mass to the reproductive biomass.

To study the effects of residence time and invasion status on flower visitors, at the peak of the flowering season the number of flower visitors were observed for the available flowering individuals (only for the treatment where flower visitors had free access) as a proxy for pollinator visits. On three sunny days, each plant was observed for five minutes (as in other studies, e.g., [63]), from 10 a.m. until midafternoon (one individual at a time, choosing individuals randomly), and number and type of each insect that touched its flower was noted. If the same insect touched the same flower (or flowers, depending on species) repeatedly, that was counted only as one visit. 

To study the effects of pollinator exclusion on seed germination with the germination trials in the greenhouse we noted the day of germination and how many seeds germinated for each individual and treatment.

### 4.4. Data Analysis

Data analysis was carried out with R v3.3.2 (R Development Core Team, 2016). 

To address the first question on the relationship between the number of flower visitors and residence time of the species, a generalized linear mixed model (GLMM) approach was employed with a Poisson distribution using the glmer function in the package lme4 [64], where in one model the effect of log(MRT) and in an alternative model the effect of invasion status was tested against the number of flower visitors. Species, individual and date (three days observations were carried out) were used as random effects on the intercept. 

To assess whether there are differences in reproductive output between treatment (factor with two levels: With pollinators/without pollinators) and invasion status (factor with four levels: Casual neophyte/established neophyte/archaeophyte/native) we used linear mixed effects models (LMM) (function lmer in lme4 package, [64]). First, an LMM was run to ensure there were no significant initial height differences between the two treatments at the beginning of the experiment, where species was used as a random effect on the intercept. As measures of reproductive output, log(total reproductive biomass + 0.1), log(number of capitula + 1), log(biomass ripe seeds + 0.1) and log(number of ripe seeds + 1) were then used as response variables, and invasion status, treatment and their interaction as explanatory variables, with species included as a random effect. If the interaction was not significant, it was removed from the model before testing main effects of status and treatment using likelihood ratio tests. Furthermore, log-transformed final height and day of first flowering and seed ripening were also analyzed in the same fashion.

To address the third question on the effect of the presence/exclusion of flower visitors on seed germination we employed an LMM to test the treatment effect on day of first germination and a GLMM with a binomial distribution to test the proportion of germinated seeds, using species as a random effect. 

Lastly, for the fourth question of how residence time and auto-fertility affect range size, we calculated the ratio of the mean treatment effect without pollinators/with pollinators for ripe seed biomass as a proxy for pollinator dependence. We then tested its effect and the effect of log(MRT) on logit-transformed ranges size in Germany using a linear model.

## 5. Conclusions

Our common garden experiment that used multiple annual Asteraceae plant species demonstrates that pollinator dependence does not play a crucial role in invasion success, with most plants along an alien–native continuum being auto-fertile to some degree. Nevertheless, established neophytes and natives seem to have higher auto-fertility abilities while simultaneously being less attractive to flower visitors. In contrast, casual neophytes and archaeophytes attract more potential pollinators. While the established neophytes’ strategy results in the largest number of ripe seeds, this does not necessarily translate into large-scale invasion success, as the ability to self-fertilize was not associated with range size. Nevertheless, the different strategies of casual neophytes, which have not yet successfully invaded, and established neophytes, hint at the possibility that with inclusion of a larger range of species auto-fertility may become an important trait of invasiveness. A better understanding of the drivers of invasiveness and the roles alien species play in native communities and networks is crucial for conservation. 

## Figures and Tables

**Table 1:** Likelihood ratio tests for final height, number of seeds, seed mass, reproductive biomass, number of capitula, flowering day and day of seed ripening tested against invasion status, treatment and their interaction. If the interaction was not (marginally) significant, it was removed from the model and then the main effects tested. (Marginally) significant values are indicated in bold characters.

Effect	Final Height	No. Seeds	Seed Mass	Reproductive Biomass	No. Capitula	Flowering Day	Seeding Day
Invasion status	χ^2^_(3 df)_ = 1.734 *p* = 0.629			χ^2^_(3 df)_ = 3.755 *p* = 0.289	χ^2^_(3 df)_ = 2.289 *p* = 0.514	χ^2^_(3 df)_ = 2.621 *p* = 0.454	χ^2^_(3 df)_ = 11.746 *p* = 0.008
Treatment	χ^2^_(1 df)_ = 15.98 *p* < 0.001			χ^2^_(1 df)_ = 0.4740 *p* = 0.491	χ^2^_(1 df)_ = 0.513 *p* = 0.474	χ^2^_(1 df)_ = 0.933 *p* = 0.334	χ^2^_(1 df)_ = 0.839 *p* = 0.360
Status × Treatment	χ^2^_(3 df)_ = 2.322 *p* = 0.508	χ^2^_(3 df)_ = 7.14 *p* = 0.068	χ^2^_(3 df)_ = 6.31 *p* = 0.097	χ^2^_(3 df)_ = 0.05 *p* = 0.997	χ^2^_(3 df)_ = 0.534 *p* = 0.911	χ^2^_(3 df)_ = 0.225 *p* = 0.974	χ^2^_(3 df)_ = 0.948 *p* = 0.814

## Supplementary Materials

The following are available online at https://www.mdpi.com/2223-7747/8/4/108/s1, Table S1: List of the study species with invasion status, minimum residence time, German range size, and seed sources. Treatments in which species flowered and produced seeds are indicated, with respective number of individuals (out of total number of individuals); Table S2: Number and type of potential pollinators that visited plants depending on invasion status; Table S3: Percentage of germinated seeds in the treatment where pollinators had free access, were excluded and totalled; Figure S1: Starting and final height depending on treatment; Figure S2: Number of ripe seeds depending on invasion status; Figure S3: Seed ripening day (day after transplanting) depending on invasion status; Figure S4: Range size in Germany depending on invasion status; Figure S5: Phylogenetic relationships among species.
