# Supplementary material for: Effects of Residence Time, Auto-Fertility and Pollinator Dependence on Reproductive Output and Spread of Alien and Native Asteraceae
Source: Plants (Basel). 2019 Apr 23;8(4):108. doi: 10.3390/plants8040108 (PMC6524061; doi:10.3390/plants8040108)
Supplement: Supplementary file 1 [file plants-08-00108-s001.pdf]

SUPPLEMENTARY MATERIALS

Effects of residence time, auto-fertility and pollinator dependence on reproductive output and spread of alien and native Asteraceae

Anna Corli[1,2], Christine S. Sheppard[1]*

[1] Institute of Landscape and Plant Ecology, University of Hohenheim, August-von-Hartmann Str. 3, 70599 Stuttgart, Germany, christine.sheppard@uni-hohenheim.de

[2] Department of Earth and Environmental Sciences, University of Pavia, Pavia, Via S. Epifanio, 14, 27100 Pavia, Italy, anna.corli01@universitadipavia.it

* Correspondence: christine.sheppard@uni-hohenheim.de

Table S1. List of the study species with invasion status, minimum residence time (MRT), German range size (%), and seed sources (name of botanical gardens or collected from wild populations in Baden-Württemberg [WP]). Treatments in which species flowered and produced seeds are indicated, with respective number of individuals (out of total number of individuals).

Species	Status	MRT	Range size Germany (%)	Seed Source	Treatment Flowered	No. flowered with pollinators	No. flowered without pollinators	Treatment produced seeds	No. produced seed with pollinators	No. produced seed without pollinators
Anthemis arvensis	Archaeophyte	2800	9.2422	WP	With/Without	5/5	5/5	With/Without	5/5	5/5
Anthemis cotula	Archaeophyte	2000	0.0920	Hohenheim	With/Without	5/5	5/5	With/Without	4/5	4/5
Artemisia annua[1]	Established neophyte	131	1.4052	Dresden	With/Without	5/5	5/5	NA	0/5	0/5
Bidens ferulifolia[2]	Casual neophyte	7	21.0940	Hohenheim	With/Without	3/5	3/5	NA	0/5	0/5
Bidens pilosa[2]	Casual neophyte	90	51.9990	Dresden	With/Without	5/5	5/5	With/Without	4/5	1/5
Calendula arvensis	Archaeophyte	2500	1.6979	WP	With/Without	4/5	2/5	With/Without	2/5	1/5
Calendula officinalis[1]	Casual neophyte	516	2.1245	WP	With/Without	3/5	4/5	With/Without	1/5	3/5
Callistephus chinensis[2]	Casual neophyte	85	0.8280	WP	With/Without	4/5	3/5	With/Without	2/5	3/5
Carthamus lanatus[2]	Casual neophyte	120	0.6440	Berlin	With/Without	5/5	5/5	With/Without	4/5	4/5
Carthamus tinctorius	Casual neophyte	154	0.1087	Hohenheim	With/Without	3/5	5/5	With/Without	3/5	2/5
Centaurea diffusa	Established neophyte	140	4.1653	WP	NA	0/5	0/5	NA	0/5	0/5
Centaurea solstitialis	Established neophyte	186	1.7397	Dresden	With/Without	5/5	5/5	With/Without	5/5	3/5
Cosmos bipinnatus[2]	Casual neophyte	32	0.1087	WP	With/Without	5/5	5/5	With	1/5	0/5
Cota austriaca	Established neophyte	133	2.3921	Hohenheim	With/Without	2/5	4/5	With/Without	2/5	4/5
Crepis capillaris	Archaeophyte	1700	5.0686	WP	With/Without	1/5	4/5	With/Without	1/5	4/5
Crepis pulchra	Native	10000	74.7574	Dresden	With/Without	5/5	5/5	With/Without	5/5	5/5
Crepis setosa	Established neophyte	154	3.2620	Dresden	With/Without	4/5	5/5	With/Without	4/5	4/5
Dittrichia graveolens	Established neophyte	66	0.5688	WP	With/Without	5/5	5/5	With/Without	5/5	5/5
Erigeron annuus	Established Neophyte	216	67.4640	WP	NA	0/5	0/5	NA	0/5	0/5
Erigeron canadensis	Established neophyte	316	1.9488	WP	NA	0/5	0/5	NA	0/5	0/5
Erigeron sumatrensis	Established neophyte	24	86.9856	WP	NA	0/5	0/5	NA	0/5	0/5
Galinsoga parviflora	Established neophyte	218	39.7123	WP	With/Without	5/5	5/5	With/Without	4/5	4/5
Galinsoga quadriradiata	Established neophyte	166	76.3299	WP	With/Without	5/5	5/5	With/Without	5/5	4/5
Glebionis coronaria	Casual neophyte	103	2.2165	WP	With/Without	5/5	4/5	With/Without	2/5	3/5
Glebionis segetum	Archaeophyte	1300	13.7755	WP	With/Without	4/5	5/5	With/Without	3/5	5/5
Guizotia abyssinica[1]	Casual neophyte	90	93.0244	Bonn	With/Without	4/5	2/5	NA	0/5	0/5
Helminthotheca echioides	Archaeophyte	6000	5.7879	WP	With/Without	3/5	3/5	With/Without	2/5	2/5
Hypochaeris glabra[2]	Native	10000	2.9023	Dresden	With/Without	5/5	5/5	With/Without	5/5	5/5
Iva xanthiifolia[2]	Established neophyte	156	24.3476	Dresden	NA	0/5	0/5	NA	0/5	0/5
Lactuca serriola	Archaeophyte	1700	8.5898	WP	NA	0/5	0/5	NA	0/5	0/5
Lactuca.virosa[2]	Archaeophyte	5000	0.2426	Berlin-Dahlem	NA	0/5	0/5	NA	0/5	0/5
Lapsana communis	Native	10000	0.5520	WP	NA	0/5	0/5	NA	0/5	0/5
Matricaria chamomilla	Archaeophyte	4100	5.1020	WP	With/Without	4/5	3/5	With/Without	2/5	1/5
Matricaria discoidea	Established neophyte	164	80.2191	WP	With/Without	3/5	3/5	NA	0/5	0/5
Pulicaria vulgaris[2]	Native	10000	69.1034	WP	With/Without	5/5	5/5	With/Without	5/5	4/5
Rudbeckia hirta[1]	Established neophyte	156	91.6611	Hohenheim	NA	0/5	0/5	NA	0/5	0/5
Sanvitalia procumbens[2]	Casual neophyte	8	5.1271	Dresden	With/Without	4/5	4/5	Without	0/5	1/5
Senecio viscosus	Native	12000	72.2566	WP	With/Without	4/5	5/5	With/Without	3/5	5/5
Senecio vulgaris	Archaeophyte	4100	90.9502	WP	With/Without	5/5	5/5	With/Without	5/5	5/5
Sonchus asper	Archaeophyte	6000	86.9354	WP	With/Without	4/5	5/5	With/Without	3/5	4/5
Sonchus oleraceus	Archaeophyte	7500	89.2104	WP	With/Without	5/5	4/5	With/Without	4/5	3/5
Tripleurospermum inodorum	Archaeophyte	2000	84.1669	WP	With/Without	1/5	3/5	NA	0/5	0/5

According to the German floristic databaseFlorKart, BfN and NetPhyD Netzwerk Phytodiversität Deutschlands e.V. (www.deutschlandflora.de), which documents species' occurrence per grid cell of 10 x 6 arc minutes (ca. 11 km x 11 km), all species occur in the raster field (no. 7221) where the common garden is located, except for the species indicated in the Table: [1]Species only occurs in one or several of the neighbouring eight raster fields; [2]indicates the ten species that do not occur in any of the nine rasters surrounding the common garden.

Table S2. Number and type of potential pollinators that visited plants depending on invasion status.

**Species**	**Sample size**	**Flies**	**Bees**	**Bumblebees**	**Total**
Casual neophytes	79	7	22	1	30
Established neophytes	64	3	3	0	6
Archaeophytes	56	17	23	1	41
Natives	47	3	1	0	4
**Total**	246	30	49	2	81

Table S3. Percentage of germinated seeds in the treatment where pollinators had free access, were excluded and total. Species which germinated in both treatments are highlighted in bold character.

**Species**	**With pollinators (%)**	**Without pollinators (%)**	**Total (%)**
Anthemis arvensis	6.00	8.10	3.80
Anthemis cotula	0	17.39	16.67
Bidens ferulifolia	0	0	0
Bidens pilosa	65.00	54.35	61.10
Calendula arvensis	5.00	0	5.00
Calendula officinalis	5.00	6.67	6.00
Callistephus chinensis	8.33	10.00	9.17
Carthamus lanatus	45.27	98.14	64.42
Carthamus tinctorius	79.17	61.53	72.97
Centaurea solstitialis	14.60	0	8.12
Cosmos bipinnatus	15.50	0	15.50
Cota austriaca	0	0	0
Crepis capillaris	0	0	0
Crepis pulchra	22.00	15.00	18.89
Crepis setosa	1.25	2.50	1.87
Dittrichia graveolens	0	0	0
Galinsoga parviflora	0	20.00	10.00
Galinsoga quadriradiata	0	0	0
Glebionis coronaria	0	0	0
Glebionis segetum	2.50	0	0.83
Hypochaeris glabra	13.00	2.50	7.50
Pulicaria vulgaris	0	0	0
Senecio viscosus	0	0	0
Senecio vulgaris	20.00	36.36	34.69
Sonchus asper	0	3.00	2.50
Sonchus oleraceus	0	0	0

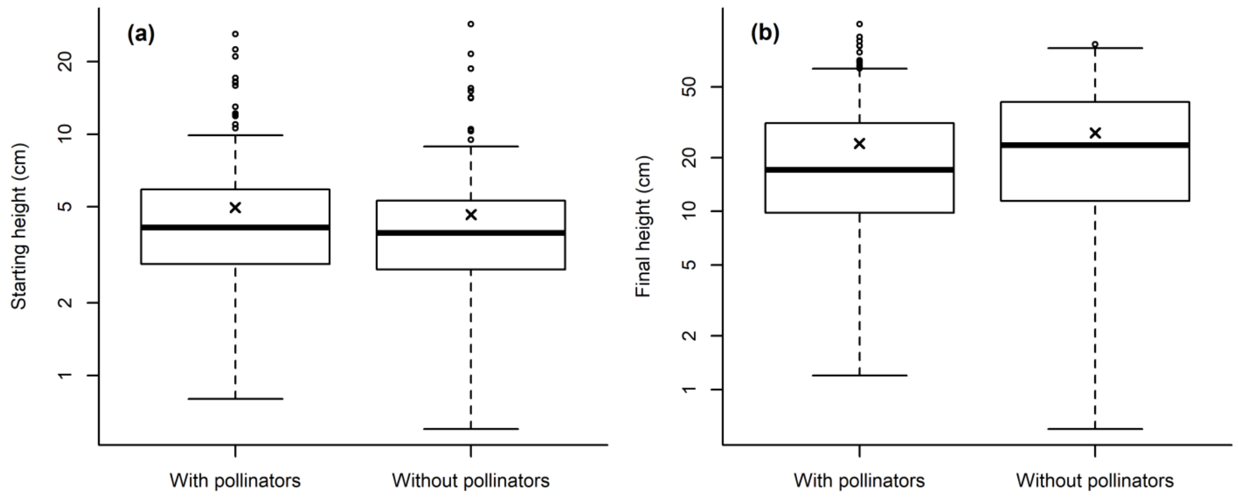

Figure S1. (**a**) Starting height: and (**b**) and final height depending on treatment for 42 Asteraceae species (397 individuals). Axes are shown on a log-scale. The boxplots show the median per group (solid line), crosses the mean, the boxes represent the 25th and 75th quantile, whiskers the normal data range and circles the outliers.

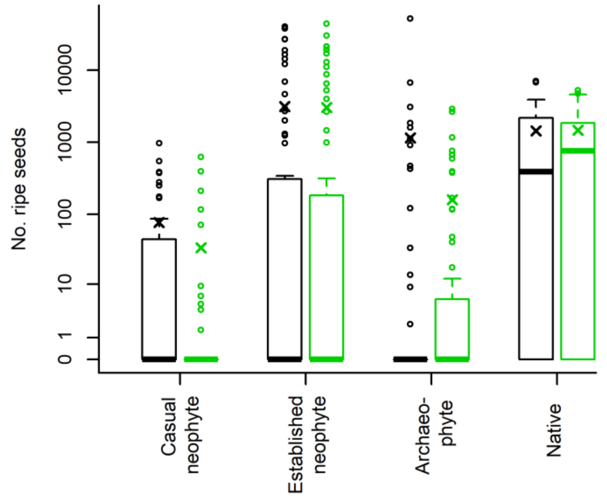

Figure S2. Number of ripe seeds depending on invasion status for 42 Asteraceae species (397 individuals). Treatment “with pollinators” in black, treatment “without pollinators” in green. The boxplots show the median per group (solid line), crosses the mean, the boxes represent the 25th and 75th quantile, whiskers the normal data range and circles the outliers. Axis is shown on a log-scale.

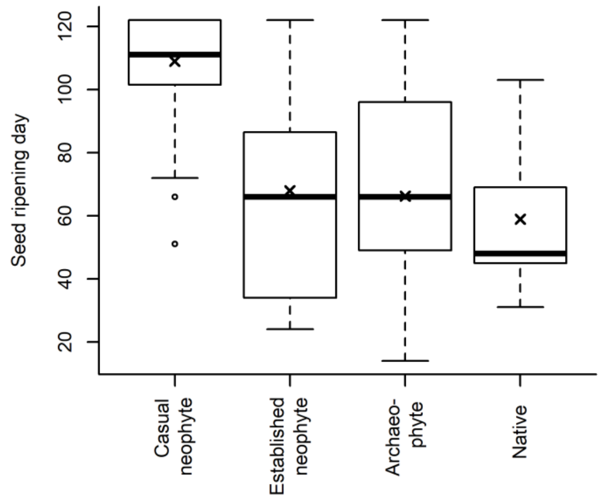

Figure S3. Seed ripening day (day after transplanting) depending on invasion status for 42 Asteraceae species (198 individuals that developed seeds). The boxplots show the median per group (solid line), crosses the mean, the boxes represent the 25th and 75th quantile, whiskers the normal data range and circles the outliers.

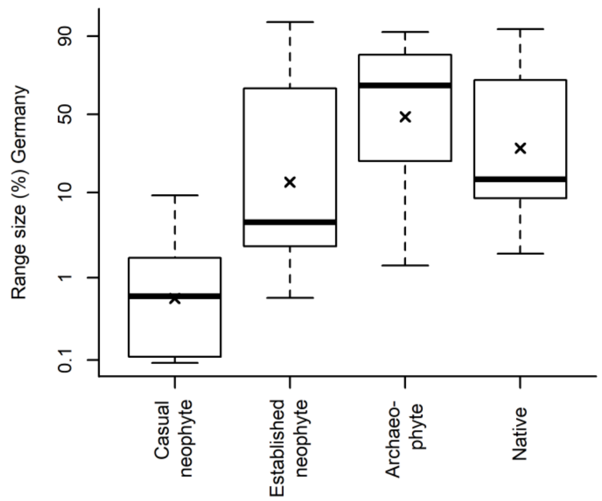

Figure S4. Range size in Germany depending on invasion status. The boxplots show the median per group (solid line), crosses the mean, the boxes represent the 25th and 75th quantile, and whiskers the normal data range (no outliers). Axis is shown on a log-scale.

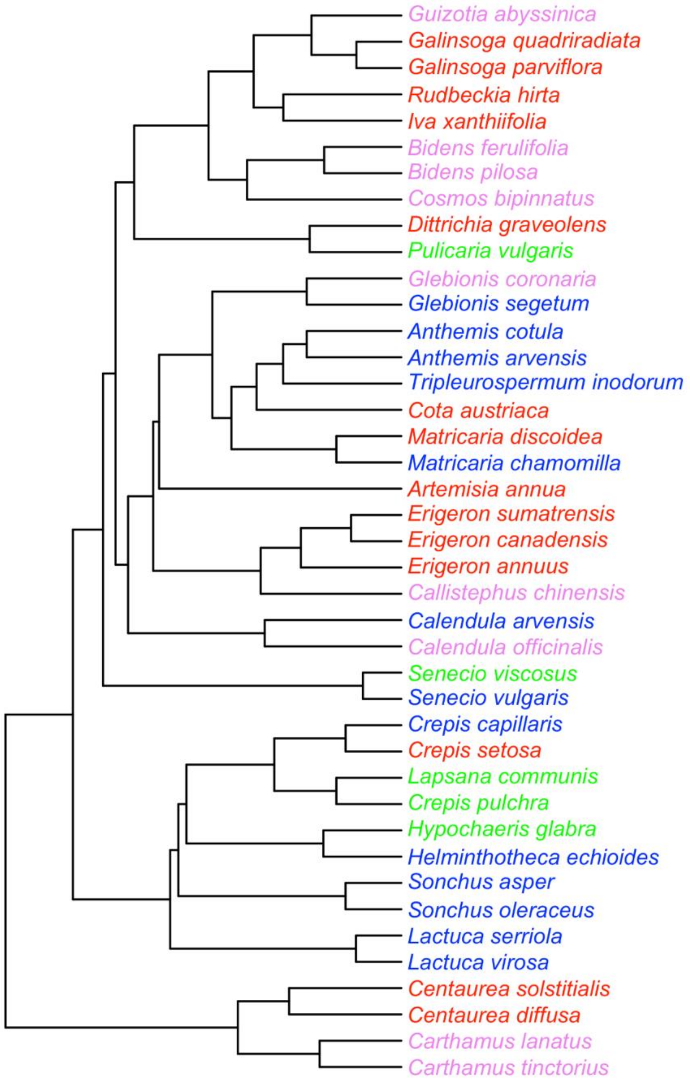

Figure S5. Phylogenetic relationships among species. Native species are indicated in green, archaeophytes blue, established neophytes red, and casual neophytes pink.
